# The structured variability of finger coordination in daily tasks

**DOI:** 10.1186/1471-2202-12-S1-P102

**Published:** 2011-07-18

**Authors:** Jovana J Belić, A Aldo Faisal

**Affiliations:** 1 Faculty of Electrical Engineering, University of Belgrade, Belgrade, 11000, Serbia; 2 Department of Bioengineering, Imperial College London, London, SW7 2AZ, UK; 3 Department of Computing, Imperial College London, London, SW7 2AZ, UK

## 

The only way we can interact with the world is through movement, and our hands are our primary mode of interaction with objects and other individuals. Understanding the neural computations underlying hand motor control in real-life situations are critical, both for the development of dexterous neuroprosthetic hands, as well as studying a defining element of human nature. Here, we take the view that motor behaviour can be understood by identifying a simplicity, which may reflect upon the underlying mechanism. Yet, the analysis of movements, and specifically hand movements is complicated by the highly variable nature of behaviour [1]. However, instead of averaging variability out we take the view that the structure of variability may contain valuable information about the task being performed [2,3].

We therefore asked 7 subjects between 20-30 years of age, to interact in 17 daily-life situations with defined objects (such as opening the door using a key, handling a fork, stacking plates, opening a drawer or reading the newspapers), each actions was repeated ten times. We collected object manipulation data using a calibrated CyberGlove I data glove (Cyberglove Systems, San Jose (CA)) that recorded the angles of the joints of each digit and the abduction angles between digits, 15 angles in total sampled at 80 Hz [3]. To extract the structure of hand configuration variability data stream we used PPCA (Probabilistic Principle Component Analysis), which built a generative model of hand configurations for each task [4]. PPCA revealed for all 17 behaviors that hand motor control restricted hand configurations on a low dimensional subspace of 4-5 dimensions (which explained 80-95% of the variance in the data for teach action), in line with previous data on evolutionary relevant hand behavior (crafting of flintstone tools) [3] and not-annotated long-term statistics of joint velocities [2].

Importantly, the low-dimensional subspaces appeared numerically distinct from each other for individual actions. This suggest the hypothesis, that specific tasks may engage specific sets of motor controllers, which orchestrated actions result in the overall behavior and produce movement variability in characteristic sub-spaces. If this is the case, then we should be able to infer the task the hand is engaged in by observing some initial portion of the finger movement data. To this end we exploit that PPCA yields us for a given set of hand configurations the likelihood that it was generated by each task. We simply predict task based on the task with the highest PPCA likelihood. Note, that PPCA ignores the temporal structure of movements (in fact the results of PPCA will be the same if the data in each trial is randomly shuffled in time) – thus correct classification relies on the sub-space of finger movement variability alone. For each of the seventeen activities parameters that characterize its distribution were determined. Half of the data were used to find the PPCA parameters, while the other half was used for testing. Using only the first 4 PPCA components in the process of classification the success of classification was 83.53% (across all tasks, classification performance of 90.25% using all 15 PPCA components). Crucially, the classification performance was about 7 times higher then chance performance for only the first 0.06% of hand configuration samples for each task. Thus, observing the first approx. 200 *ms* of hand configurations (from our identical starting positions) was sufficient to characterize the entire hand task. This supports the view that motor cortex organises behaviour in a low-dimensional manner to avoid the curse of dimensionality in terms of computational complexity. This predictability could be exploited to build smart, context-sensitive neuroprosthetics controllers that infer the task “at hand” based on the movements of remaining fingers or the activity of finger muscles in the arm stump.
